# Effect of Atmospheric Pressure Plasma Jet Treatments on Magnesium Phosphate Cements: Performance, Characterization, and Applications

**DOI:** 10.1021/acsbiomaterials.3c00817

**Published:** 2023-11-20

**Authors:** Rita Gelli, Monica Tonelli, Francesca Ridi, Dominik Terefinko, Anna Dzimitrowicz, Pawel Pohl, Aleksandra Bielawska-Pohl, Piotr Jamroz, Aleksandra Klimczak, Massimo Bonini

**Affiliations:** † Department of Chemistry “Ugo Schiff” and CSGI, University of Florence, via della Lastruccia 3, 50019 Sesto Fiorentino, Florence, Italy; ‡ Department of Analytical Chemistry and Chemical Metallurgy, Wroclaw University of Science and Technology, Faculty of Chemistry, 27 Wybrzeze Wyspianskiego, 50-370 Wroclaw, Poland; § Hirszfeld Institute of Immunology and Experimental Therapy, Polish Academy of Sciences, The Laboratory of Biology of Stem and Neoplastic Cells, 12 R. Weigla, 53-114 Wroclaw, Poland

**Keywords:** bone cements, cold atmospheric pressure plasma, roughness, scaffolds, mesenchymal cells, biocompatibility

## Abstract

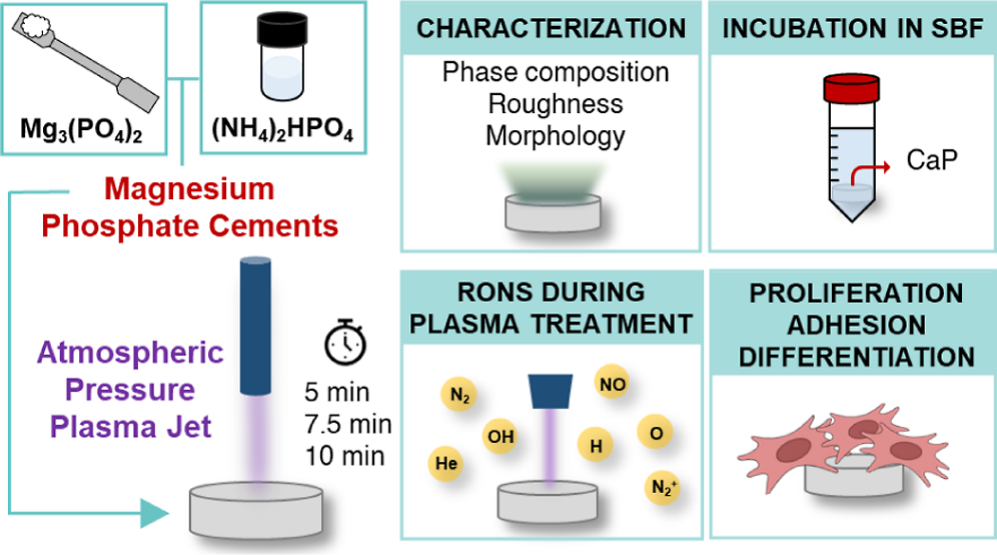

Atmospheric pressure plasma treatments are nowadays gaining importance to improve the performance of biomaterials in the orthopedic field. Among those, magnesium phosphate-based cements (MPCs) have recently shown attractive features as bone repair materials. The effect of plasma treatments on such cements, which has not been investigated so far, could represent an innovative strategy to modify MPCs’ physicochemical properties and to tune their interaction with cells. MPCs were prepared and treated for 5, 7.5, and 10 min with a cold atmospheric pressure plasma jet. The reactive nitrogen and oxygen species formed during the treatment were characterized. The surfaces of MPCs were studied in terms of the phase composition, morphology, and topography. After a preliminary test in simulated body fluid, the proliferation, adhesion, and osteogenic differentiation of human mesenchymal cells on MPCs were assessed. Plasma treatments induce modifications in the relative amounts of struvite, newberyite, and farringtonite on the surfaces on MPCs in a time-dependent fashion. Nonetheless, all investigated scaffolds show a good biocompatibility and cell adhesion, also supporting osteogenic differentiation of mesenchymal cells.

## 1 Introduction

Magnesium-based biomaterials are nowadays at the forefront in the development of new materials for orthopedic applications due to their biocompatibility and biodegradability.^1−4^ Among them, magnesium phosphate cements (MPCs) are recently attracting a large interest in the field of bone cements, which are defined as biomaterials obtained from the mixing of a powder and a liquid phase that can be molded and implanted as a paste and set within the body.^5^ In comparison to the well-established calcium phosphate cements (CPCs), it was recently reported that MPCs can display a better combination of strength, setting time, and resorption rate than CPCs while remaining biocompatible.^6,7^ MPCs are obtained from the reaction of MgO or Mg_3_(PO_4_)_2_ with an aqueous solution of a phosphate-based salt such as (NH_4_)_2_HPO_4_, NaH_2_PO_4_, K_2_HPO_4_, and H_3_PO_4_: after the initial paste formation, a hard and compact material forms due to crystals entanglement. Depending on the precursors used, the cement binding phase can be constituted by a variety of phases, the most important being struvite MgNH_4_PO_4_·6H_2_O, K-struvite MgKPO_4_·6H_2_O, and newberyite MgHPO_4_·3H_2_O.^7^ MPCs can be used directly as pastes to fill bone voids and stabilize fractures or to prepare implantable bioceramic scaffolds with a customizable shape. Their most attractive features include fast hardening, high adhesive and early strength, good mechanical properties, an appropriate resorption rate, and biocompatibility. The resorption aspect is particularly important for bone cements, as such materials are expected to slowly degrade in the body after implantation, providing support to the bone tissue in the initial stages but leaving room for the new bone tissue formation by osteoblasts. In this context, it was demonstrated that MPC-based implants are entirely resorbed over time maintaining the structural stiffness^7,8^ and, in comparison to CPCs, a higher resorption rate and the enhanced bone regeneration were often reported.^9−11^ Moreover, MPCs also play a vital role in the bone metabolism for healing as their degradation products stimulate the osteogenesis and bone defect repair.^6^ The interest in MPCs toward biomedical applications is rapidly growing,^12−18^ and various strategies have been recently developed to further improve MPCs’ features, such as the inclusion of polymeric additives to enhance the injectability and printability^19,20^ or the addition of porogens and templating agents to the formulation to induce the presence of macroporosity in the cement matrix.^15,21,22^

Along with the modifications of the bulk material, the cement surface is of great importance, as it represents the first region that cells encounter when they get in contact with the material, being crucial for their adhesion, spreading, and ultimately for the material biocompatibility.^23^ Surface modifications aim at creating a specific chemical and physical environment that offers a favorable cellular response in tissues and include changes in topography and morphology, functionalization, and coatings.^23^

A technology that has recently received great attention for the improvement of biomaterials surfaces is cold atmospheric pressure plasma (CAPP).^24−26^ CAPP, also referred to as “non-thermal” or “non-equilibrium” plasma, consists of a partially ionized gas and can be operated in an open environment, at ambient temperature and pressure, reaching less than 40 °C in the application site.^27^ CAPP treatments have already been used to modify the surface properties of materials (wettability, chemical composition, adhesion, among others), as well as to inactivate pathogens in the food industry, agriculture, and medicine.^28^ In this context, CAPP treatments on MPCs might be a promising strategy not only to sterilize them before clinical application but also, in principle, to improve their bioactivity and surface properties. In addition, the possibility of confining the plasma glow in small regions of few mm using for instance pen-like devices allows for the design of patterned surfaces, which are of interest to control the behavior of cells.^29−31^

In the field of phosphate-based scaffolds, CAPPs treatments were used to improve the hydrophilicity and osteoconductivity of calcium hydroxyapatite ceramics,^32^ to increase the cell attachment and proliferation on a hydroxyapatite/tricalcium phosphate scaffold,^33^ to treat dentin,^34,35^ to improve the osteogenic differentiation of human bone marrow mesenchymal stem cells on nanohydroxyapatite/chitosan scaffolds,^36^ and to modulate antibiotic release from tricalcium phosphate ceramics.^37^ In light of those results, magnesium phosphate-based scaffolds could also benefit from the application of CAPP treatments to improve their surface properties in terms of cell interaction and, in turn, of their biocompatibility once implanted in the body. In addition, the phases typically constituting MPC are often hydrated and labile,^38,39^ so it might be interesting to understand from a fundamental perspective if highly energetic treatments such as plasma-based ones affect the phases composition on the surface, and eventually the biocompatibility of the material.

The goal of this work is to modify the surface properties of MPCs by the application of the CAPP to improve their interaction with cells. Cements were prepared and plasma-treated at different times. The reactive oxygen and nitrogen species (RONS) produced during the treatment were determined, and the modifications induced by CAPP were analyzed by combining different experimental techniques to unravel the changes in the phases, the morphology, and the roughness of their surfaces. The bioactivity of the MPCs was confirmed with a test in simulated body fluid (SBF), while the biocompatibility toward human mesenchymal stem cells was assessed in terms of proliferation, osteogenic differentiation, and adhesion.

## 2 Materials and Methods

### 2.1 Reagents

Magnesium hydroxide (Mg(OH)_2_, purity > 95%), KCl (purity > 99%), Na_2_SO_4_·10H_2_O (purity > 99%), and TRIS [tris(hydroxymethyl)aminomethane, purity > 99.7%] were purchased from Fluka. Newberyite (MgHPO_4_·3H_2_O, purity > 97%), NaCl (purity > 99.5%), MgCl_2_·6H_2_O (purity > 99%), and CaCl_2_ (purity > 93%) were obtained from Sigma-Aldrich. NaHCO_3_ (purity > 99.5%) and K_2_HPO_4_·3H_2_O (purity > 99%) were purchased from Merck. HCl 37% concentration was obtained from Carlo Erba Reagents. Di-ammonium hydrogen phosphate [(NH_4_)_2_HPO_4_, DAHP, purity > 99%] was supplied by Riedel de Haën. Milli-Q water (resistivity 18.2 MΩ·cm) was used throughout all the experiments. The reagents for reactive oxygen species (ROS) determination such as potassium iodide (KI) and starch were bought from Avantor Performance Materials. Agar was purchased from A&A Biotechnology. All materials were used as received, without any further purification.

### 2.2 Samples Preparation

MPC samples were prepared upon reaction of trimagnesium phosphate [TMP, Mg_3_(PO_4_)_2_] and a 3.5 M aqueous solution of DAHP. TMP was obtained from the calcination of Mg(OH)_2_ with MgHPO_4_·3H_2_O, as described elsewhere.^40^ Cement samples were prepared by mixing 0.5 g of TMP with 0.333 mL of DAHP 3.5 M (powder/liquid ratio 1.5 g/mL). The reaction that takes place is the following
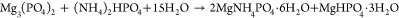


The two components were thoroughly mixed for 30 s, and the obtained paste was poured into a cylindrical mold for setting. For samples to be used for confocal Raman microscopy and SBF experiments, molds of 1.3 cm diameter and 4 mm thickness were used, while for atomic force microscopy (AFM), field emission-scanning electron microscopy (FE-SEM), and biological experiments, molds with a diameter of 0.4 cm and thickness of 1 mm were used. MPCs were set at 37 °C and relative humidity > 96% for at least 5 days before characterization.

### 2.3 Cold Atmospheric Pressure Plasma Treatment of MPC

**Figure 1:**
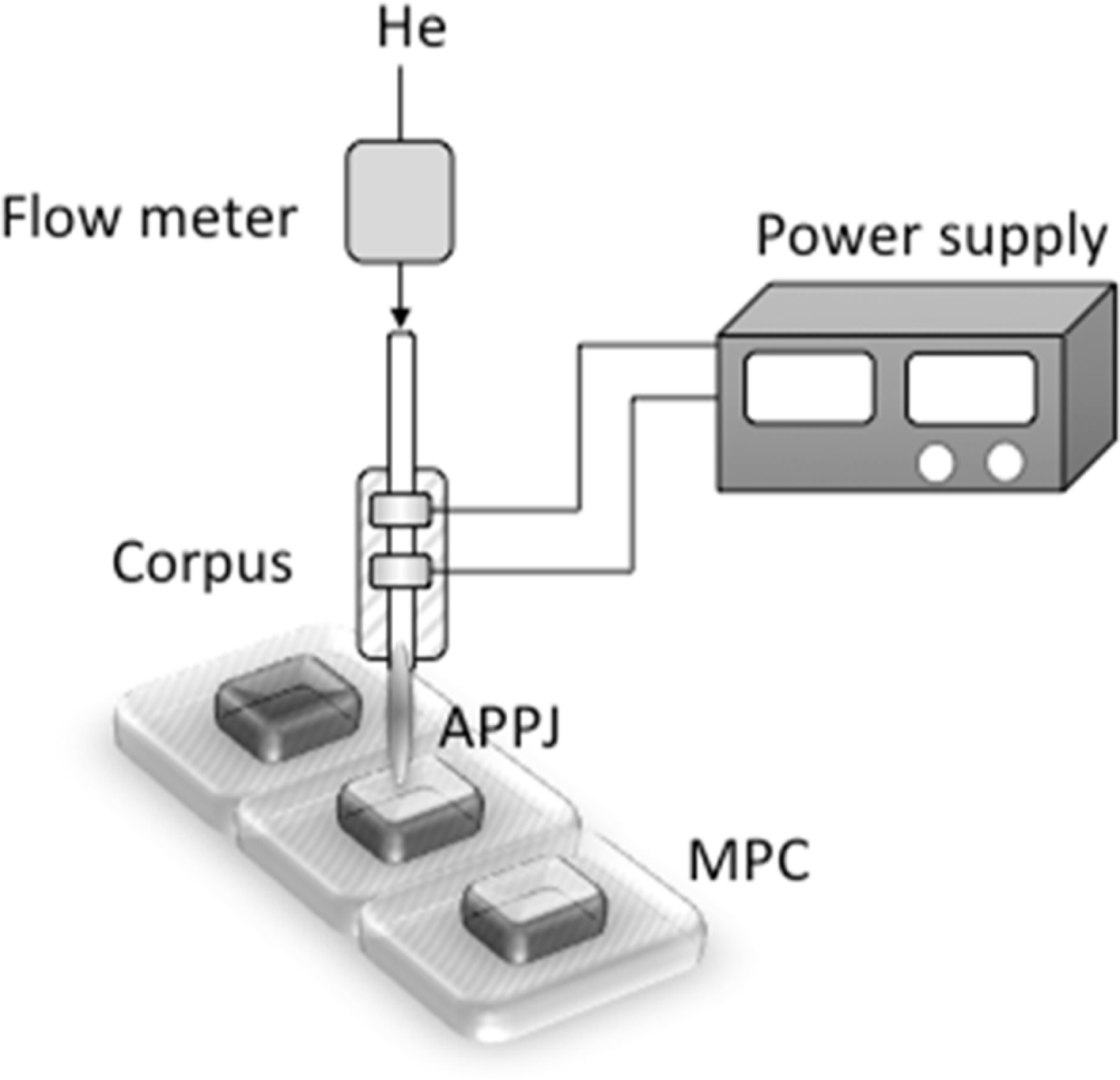
Representative layout of the CAPP-based system used for MPCs treatment. The system consists of the main corpus (with tungsten electrodes and a quartz tube), an APPJ power supply, and a flow meter for helium.

For MPCs treatment, an atmospheric pressure plasma jet (APPJ)-based reaction-discharge system was previously patented by some of the authors (polish patent no. P. 241305) was adapted and used (Figure 1).^41^ The central part of the system is a corpus, comprising the quartz tube and tungsten electrodes, immersed into an Epoxy E-57 resin. To make the corpus tangible and safe to use, it was covered by a ceramic layer. The APPJ was generated under a helium atmosphere in the dielectric barrier discharge (DBD) regime as a CAPP source. The flow rate of He was maintained constant using a mass flow meter (Tyco Electronics, Poland) and set to 1.3 L min^–1^. For APPJ operation, a proper HV potential was supplied from the DBD power supply (Dora Electronic Equipment, Poland). A plasma tip of approximately 40 mm in length formed from the end of the ceramic corpus and the MPCs were treated for 5, 7.5, or 10 min.

### 2.4 Protocol of the Test in Simulated Body Fluid

SBF was prepared according to the classical recipe from Kokubo and Takadama.^42^ The prepared volume was 0.5 L. Salts were weighed following the order reported in Table 1 and dissolved in Milli-Q water in a glass volumetric flask at 37 °C. Each salt was completely dissolved before the addition of the following one. Before the addition of the TRIS, the measured pH was about 1 (pH meter 7+ with DHS electrode, XS instruments). TRIS was slowly added to the solution while the pH was constantly monitored. After the complete TRIS addition, the pH was 7.45. HCl 1 M was added dropwise to the solution, reaching pH = 7.4 at 37 °C. SBF was slowly cooled to room temperature, transferred in a plastic bottle, and stored in the fridge.

**Table 1:** Amount of Reactants Used to Prepare 0.5 L of SBF, Together with the Final Ionic Concentrations

reagent	amount	ion	concentration (mM)
NaCl	4.0175 g	Na^+^	142.0
NaHCO_3_	0.1775 g	K^+^	5.0
KCl	0.1125 g	Mg^2+^	1.5
K_2_HPO_4_·3H_2_O	0.1155 g	Ca^2+^	2.5
MgCl_2_·6H_2_O	0.1555 g	Cl^–^	147.8
HCl 1 M	19.5 mL	HCO_3_^–^	4.2
CaCl_2_	0.1460 g	HPO_4_^2–^	1.0
Na_2_SO_4_·10H_2_O	0.0817 g	SO_4_^2–^	0.5
TRIS	3.0590 g	pH	7.40

For the test, cement samples (disks with diameter 1.3 cm, thickness 4 mm) were placed in 50 mL Falcon test tubes, with the plasma-treated surface facing the bottom of the tube, following Kokubo protocol. 50 mL of SBF preheated at 37 °C were added in each tube, and samples were incubated at 37 °C in an oven for 28 days. As a control experiment, a MPC was incubated in the same conditions but using water instead of SBF. Disks were then removed from the tube using tweezers, plunged in water to remove the unreacted salts and dried under the fume hood, at room temperature, for 3 days. Before the FE-SEM experiments, samples were kept for 3 h in a vacuum desiccator to ensure complete removal of water.

### 2.5 Characterization Techniques

#### 2.5.1 Confocal Raman Microscopy

Confocal Raman microscopy was carried out using a Renishaw InVia Qontor confocal microRaman system equipped with a front illuminated CCD camera and a research-grade Leica DM 2700 microscope. Maps were collected by using a 20× objective (WD 1.15 mm, NA 0.40) and a laser operating at 785 nm with a power of 100 mW and a grating of 1200 lines/mm. Maps were collected using the StreamLine mode. For each sample, four maps with an area of 300 μm × 200 μm each (step size: 5 μm) were collected in different regions of the surface. The exposure time for each spectrum was 2 s, with 1 accumulation, in the range 155–1355 cm^–1^. Spectra were processed with the Renishaw software WiRE, corrected for cosmic rays, baseline, and noise and then used to obtain maps.

As a reference, spectra of the pure phases constituting the samples (TMP, newberyite, and struvite) were also collected. For TMP, we analyzed the powder used as a precursor for the preparation of cements, while for newberyite, we analyzed the commercial one (see Section 2.1). Synthetic struvite was prepared as described in a previous study.^43^ The spectra were recorded using a 20× objective, 10 mW of laser power, 10 s per spectrum, and three accumulations. These spectra were used as reference spectra to perform a multivariate method component analysis integrated with WiRE software. The analysis allows for the estimation of the concentration of the phases present in the mapped area, defined as percentage values derived from the least-squares fitting (non-negative least squares method) of multiple reference spectra at all points in the analyzed area. Each map was analyzed, and the results are expressed as the average ± standard deviation of the four maps collected in each sample.

#### 2.5.2 Atomic Force Microscopy

AFM measurements were performed with a Park System XE-7 microscope equipped with a noncontact cantilever (PPP-NCHR probe, force constant 42 N/m, frequency 330 kHz) in the noncontact mode. MPCs disks were fixed to sample holders by using cyanoacrylate glue. For each sample, 10 maps of 25 × 25 μm were collected. Calculation of the roughness parameters and image processing were performed by using Park Systems XEI software. Roughness values are expressed as the average ± standard deviation of the results obtained for the 10 maps for each sample.

#### 2.5.3 Field Emission-Scanning Electron Microscopy Coupled with Energy-Dispersive X-ray Spectroscopy

FE-SEM micrographs were collected using a Zeiss ΣIGMA FE-SEM (Carl Zeiss Microscopy GmbH), with an accelerating voltage of 2.0 kV, a sample-detector distance ∼2 mm, and using the inLens detector. Cements were fixed on aluminum stubs by means of conductive tape, and the bottom was surrounded by colloidal graphite to improve conductivity.

Energy-dispersive X-ray spectroscopy (EDX) was carried out with an X-act Silicon Drift Detector (Oxford Instruments), and the spectra were recorded with an accelerating voltage of 10.0 kV, and a working distance of ∼ 8 mm. FE-SEM images coupled with EDX maps were also collected with an accelerating voltage of 10.0 kV and a sample-detector distance of 8.5 mm, using either the SE2 Detector (FE-SEM micrograph) or the X-act Silicon Drift Detector (EDX maps).

#### 2.5.4 RONS Produced during CAPP Treatment of MPCs

To qualitatively measure the RONS produced in the gaseous phase during CAPP operation, studies were carried out using optical emission spectrometry (OES). The radiation emitted by the APPJ was imaged by UV achromatic lens (*f* = 60) on the slit (10 μm) of the high-resolution Shamrock SR-500i (Andor) spectrometer, equipped with two holographic gratings (1800 and 1200 grooves per mm, for 200–400 and 400–900 nm spectral range, respectively). Additionally, the Newton DU-920 CCD camera (Andor), working in Full Vertical Binding (FVB) mode, was applied. The OES spectra were accumulated, and 10 spectra with an integration time 0.1 s were measured. The Solis S software (Andor) was applied for the acquisition and processing.

To quantitatively determine the ROS produced during the CAPP treatment of MPCs, their spatial distribution following irradiation of the APPJ was visualized using gel models, prepared from the mixture of potassium iodide [KI, 0.3% (m/v)], starch [C_6_H_10_O_5_, 0.5% (m/v)], and bacteriological agar in two different concentrations [1.2 and 2.0% (m/v)]. The reagents were suspended in 200.0 mL of deionized water and heated to 70 °C, under magnetic stirring to obtain a homogeneous solution. The so-obtained solution was portioned into two types of containers: plastic Petri dishes and 20 mL glass vials. Containers filled with prepared solutions were further left to solidify the gels. Obtained gels were placed 7.00 mm under the plasma tip with the aid of a digital caliper and treated with APPJ for 5 min, 7.5 min, or 10 min. As a result of the interaction between CAPP and prepared KI-starch gels, the appearance of navy blue color was observed. This was linked to the presence of ROS (especially ^•^OH, O, O_3_, H_2_O_2_, and HO_2_) produced by the used APPJ, which poses an oxidative potential exceeding 0.54 V for I^–^ ions.

### 2.6 Biocompatibility of MPCs with Human Mesenchymal Stem Cells

#### 2.6.1 Cell Culture

The Human Adipose Tissue Mesenchymal Stem Cell line (HATMSC2) has been established in the Laboratory of Biology of Stem and Neoplastic Cells IITE PAS using the hTERT and pSV3-neo plasmids, from primary MSCs isolated from adipose tissue, as introduced in previously described protocol.^44^ Subsequent characterization of the HATMSC2 cell line confirmed the phenotype of primary MSCs: CD73+, CD90+, CD105+ and negativity for hematopoietic markers CD34– and CD45–. The HATMSC2 cells were cultured in DMEM supplemented with 10% fetal bovine serum (Gibco, Thermo Scientific), a 100 U mL^–1^ penicillin/100 μg mL^–1^ streptomycin solution (Gibco, Thermo Scientific), and l-glutamine (Gibco, Thermo Scientific) until reached confluence and used for further experiments to assess proliferation activity and biocompatibility with MPC scaffolds. Before the experiments were performed, the MPC samples were either left untreated or APPJ treated for 5.0 or 7.5 min (details in Section 2.3).

#### 2.6.2 Proliferation Activity of HATMSC2 Cells on MPCs

The HATMSC2 cells were seeded in the 96-well plates at a concentration of 2 × 10^3^ cells/well in DMEM without fetal bovine serum, and supplemented with 100 U mL^–1^ penicillin solution and a 100 μg mL^–1^ streptomycin solution in the presence of untreated MPC scaffold, APPJ-treated scaffold for 5 min, and APPJ-treated scaffold for 7.5 min. At the defined time point (from day 0 to day 7), 100 μL of an MTT solution (0.4 mg mL^–1^ of MTT) was added to the cells and incubated for 3 h in the dark at 37 °C. Then, the MTT solution was removed, and 100 μL of DMSO was added to each well and incubated at 37 °C for 10 min to dissolve the purple crystals. The absorbance was measured at 570 nm with a Victor 2 multifunction microplate reader (PerkinElmer). The metabolic activity was calculated as the mean value of the absorbance acquired in duplicate in two independent experiments.

#### 2.6.3 Adhesion of HATMSC2 Cells to MPCs

To assess the efficacy of adhesion of HATMSC2 cells to CAPP-treated MPC scaffolds, 5 × 10^5^ cells/well were seeded in a 24-well plate with MPC scaffolds and allowed to attach within 3 h at 37 °C. After the adhesion process the scaffolds were transferred into a new 24-well plate and NucBlue Live ReadyProbes Reagent (Hoechst 33342, Thermo Fisher) was added to each well to visualize the number of cells that attached to the biomaterial using an Axio Observer inverted microscope (Zeiss). The images were processed with the Zen Blue software (Zeiss). In the next step, the number of cells that adhered to the biomaterial was evaluated using the previously described method^45^ based on the PicoGreen protocol (Quant-iT PicoGreen dsDNA Assay Kits and dsDNA Reagents, Thermo Fisher). The number of adhered cells was calculated by considering the total number of cells and the number of cells that adhered to the plastic bottom.

#### 2.6.4 Osteogenic Differentiation of HATMSC2 Cells on MPCs

To examine the osteogenic differentiation potential of HATMSC2 cells in the presence of MPC, the cells were seeded in a 48-well plate at a density of 1 × 10^3^ cells/well and allowed to attach overnight. After overnight incubation, the culture media were changed to an osteogenic differentiation medium (PromoCell) and DMEM (control medium) for the control cells. The osteogenic medium was replaced every 3 days. After 14 days of incubation, the osteogenic differentiation potential of HATMSC2 cell line was assessed through visualization with Alizarin Red S staining. Briefly, the differentiation media were removed, and the cells were washed with PBS (IITE PAS) and fixed for 20 min at RT in a 3.7% formaldehyde (Merck) and stained with 200 μL of Alizarin Red S (Merck) for 10 min. Microscopy assessment was performed using a Primovert inverted microscope (Zeiss). To quantify the differentiation process, Alizarin red was extracted with 10% cetylpyridinium chloride (Sigma-Aldrich) after 2 h of incubation at 37 °C. Finally, the absorbance was measured at 405 nm with the Victor 2 multifunction microplate reader (PerkinElmer).

The statistical analyses related to the biological studies were performed using Prism 8.0 (GraphPad Software, USA). The comparison of the investigated groups versus the control group or untreated MPCs scaffold was made using one-way ANOVA with Dunnett’s post hoc test. Statistical significance was calculated as *p* values < 0.05.

## 3 Results and Discussion

**Figure 2:**
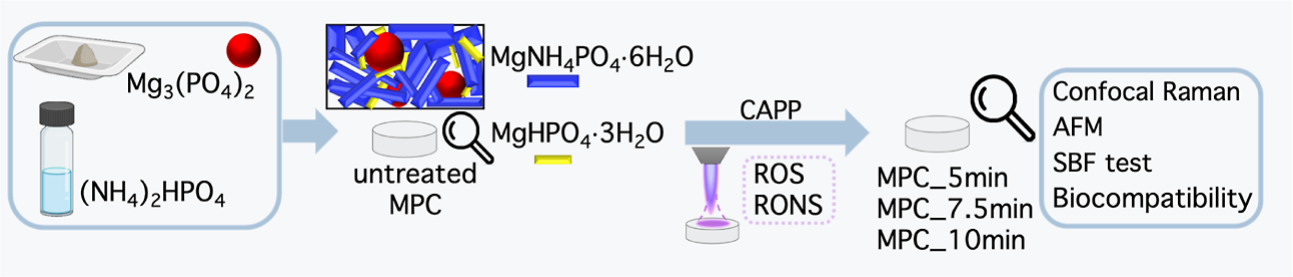
Schematic diagram of the samples’ preparation and characterization.

The samples prepared and characterized throughout this study are shown in Figure 2. The cement pastes were prepared following the protocol described in detail in Section 2.2 and cast into molds for setting. Then, samples were either left untreated or treated by APPJ (details in Section 2.3) for 5, 7.5, or 10 min. All samples were investigated by multiple physicochemical techniques to understand the effect of the plasma treatment on MPCs and, considering potential applications in the biomedical field, we thoroughly characterized their surface properties, which are of utmost importance in this context. To this purpose, the bioactivity and biocompatibility of the samples were also assessed. The results obtained from these characterizations are analyzed and comprehensively discussed in the following sections.

### 3.1 Characterization of Pristine Cements

**Figure 3:**
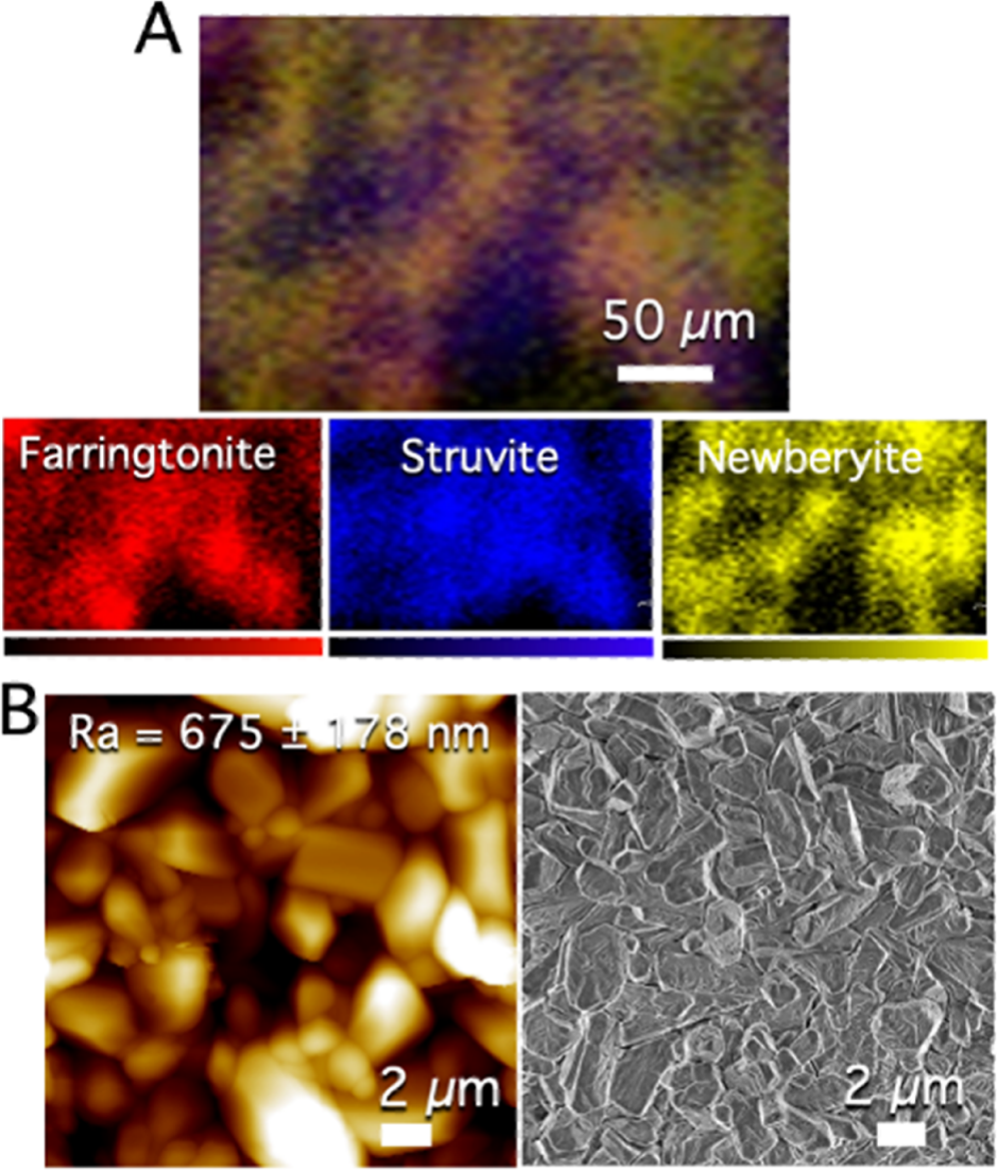
(A) Confocal Raman map of the untreated MPC sample, resulting from the overlay of the maps of the phases constituting the sample (farringtonite in red, struvite in blue, and newberyite in yellow); the Raman image was obtained by performing the component analysis integration with the software WiRE, using as references the spectra acquired on the pure farringtonite, newberyite, and struvite phases. (B) AFM topography map (left) and FE-SEM image of MPC (right). The reported arithmetic mean roughness value Ra is the average of 10 maps acquired on different regions of the samples ± standard deviation.

Untreated MPC was investigated by confocal Raman microscopy to check the composition of the surface in terms of mineral phases and by means of AFM and SEM to evaluate the topography and morphology (see Figure 3). Aiming at the detection of phase and compositional changes caused by the CAPP treatment on the surface of the cements, confocal Raman microscopy represents a unique tool: in fact, the lability of MPC samples complicates their investigation when they are exposed to vacuum and/or energetic radiations. Confocal Raman microscopy allows us to investigate the sample at room conditions. Its exposure to lasers for a very short time makes it possible to detect the phase changes taking place in the samples. Moreover, confocal Raman microscopy was recently demonstrated by some of the authors as an effective technique for the assessment of the phases forming upon hydration in MPC samples.^43^ Here, we also conducted a component analysis of the acquired maps to obtain the percentages of the phases constituting the samples. First, we collected the spectra of the pure phases expected to be present in the cements (details in Section 2.5.1), reported in Figure S1, which were used as references for the analysis of the confocal Raman maps. Figure 3A shows that the surface of untreated MPC exposes farringtonite, struvite, and newberyite phases. The white light image of the mapped area is also reported in Figure S2, together with the corresponding Raman map. It is worth mentioning that during the MPC formation, the reaction takes place through the dissolution and reprecipitation processes around the grains of TMP, i.e., farringtonite, which is in excess with respect to the other reactant (DAHP). The reaction between TMP and DAHP leads to the formation of struvite and newberyite as the reaction products, which are present in the final matrix together with unreacted TMP (see the reaction reported in Section 2.2). The quantitative analyses performed on the mapped areas (see the experimental details in Section 2.5.1) allowed us to estimate the phases’ amounts, and the results are reported in Table 2. According to the results, a mixture of these three phases is present on the MPC surface with farringtonite and struvite as the main components.

**Table 2:** Concentration Estimates from Confocal Raman Microscopy of the Phases Present in the Mapped Areas^a^

sample	farringtonite (%)	struvite (%)	newberyite (%)	Ra (nm)
MPC	44 ± 2	45 ± 1	11 ± 1	675 ± 178
MPC_5 min	72 ± 1	4 ± 1	24 ± 1	613 ± 86
MPC_7.5 min	59 ± 2	4 ± 1	37 ± 2	572 ± 62
MPC_10 min	59 ± 4	3 ± 1	38 ± 4	

a The results are expressed as the average ± standard deviation of four confocal Raman maps collected in each sample. Ra values are the average of 10 AFM maps acquired on different regions of the samples ± the standard deviation.

Figure 3B shows the topography (left) and morphology (right) of untreated MPC. The sample shows micrometric elongated structures, exposing a rough surface (Ra, arithmetic mean roughness: 675 ± 178 nm), which could be compatible with the deposition of hydroxyapatite and for biological applications,^46−49^ as demonstrated by the bioactivity and biocompatibility tests reported in Section 3.4. The FE-SEM images reported in Figures 3B and S3 in the Supporting Information confirm the heterogeneity of the MPC surface. It is possible to recognize abundant farringtonite crystals, appearing as smooth micrometric objects, and struvite crystals, characterized by a prismatic elongated structure with cross-shaped and Y-shaped cracks.^43,50^

### 3.2 Determination of RONS Produced during MPCs Treatment by APPJ

The emission spectra of APPJ were acquired for the plasma jet zone—the MPC interface zone to estimate the RONS produced during the CAPP treatment of MPCs. The representative OES spectra of APPJ are presented in Figure S4 for 200–400 nm and 400–900 nm spectral ranges. As can be seen from Figure S4A in the UV region, the OES spectrum was dominated by N_2_ (C^3^Π_u_–B^3^Π_g_ system) bands with the band heads: (2–0) at 297.8 nm, (2–1) at 313.6 nm, (1–0) at 315.9 nm, (0–0) at 337.1 nm, (1–2) at 353.7 nm, (0–1) at 357.7 nm, (2–4) at 371.0 nm, (1–3) at 375.5 nm, and (0–2) at 380.5 nm, as well as bands of the OH radical belonging to the A^2^Σ–X^2^Π system with the band heads (1–0) at 282.9 nm and (0–0) at 308.9 nm. Additionally, the numerous bands of NO (A^2^Σ^+^–X^2^Π system) were easily excited in the range 200–280 nm. The bands of the N_2_^+^ molecule (B^2^Σ^+^_u_–X^2^Σ^+^_g_) with the band heads at 391.4 nm (0–0) and at 427.8 nm (1–1) 336.0 were correspondingly identified in the spectra range 380–430 nm. It should be noted that there were O atomic lines (at 777.2 and 844.6 nm) as well as H atomic lines (at 486.1 and 656.2 nm) in the OES spectra of APPJ (see Figure S4B). The He atomic lines at 501.5, 587.56, 667.8, and 706.5 nm (with the excitation energy within 23–24 eV) were also excited.

**Figure 4:**
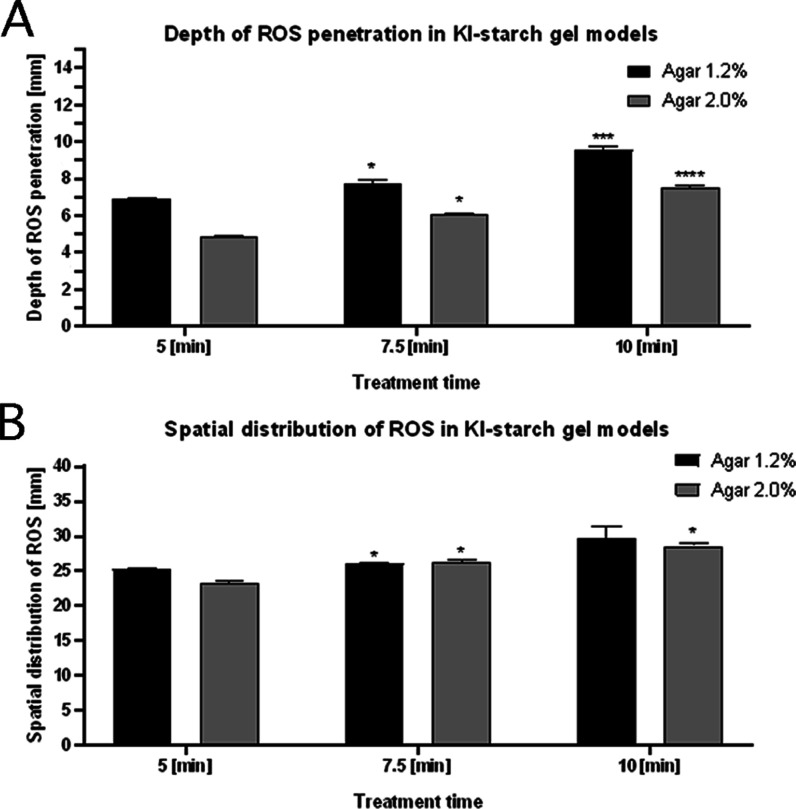
Quantitative determination of ROS. (A) The depth of the ROS penetration in the KI-starch gels following irradiation with the APPJ system for 5, 7.5, and 10 min under the selected parameters. (B) The diameter of the spatial distribution of ROS in the KI-starch gels following irradiation with the APPJ system for 5, 7.5, and 10 min under the selected parameters.

The experiments with solid gels, composed of the KI-starch mixtures and immersed in bactericidal agar, were conducted to visualize the spatial distribution of ROS, being a result of the MPC treatment by the APPJ. Two concentrations of bactericidal agars (1.2 or 2.0%) were used to verify the differences in generated navy blue regions, depending on the density of treated models. In the first experiment, the irradiated solid gels placed in the glass vials (in the central point of the vial) turned into a navy blue color that propagated into the deeper parts of the gel (Figure 4A). This allowed us to measure the penetration depth of all ROS generated by the APPJ. In more detail, with the increased treatment time of the APPJ, a significant increase in the penetration depth of ROS was observed in the case of the 1.2% agar. During the treatment time of 7.5 min it was 7.8 ± 0.2 mm (vs 6.9 ± 0.1 mm for the 5 min treatment, *p < 0.02), while for 10 min, it was 9.6 ± 0.2 mm (vs 6.9 ± 0.1 mm for the 5 min treatment, ***p < 0.0002). In the case of the 2.0% agar, lower penetration depths were determined for each APPJ treatment time. Nevertheless, a similar dependence was observed in the case of the treatment time of the APPJ on the ROS penetration depth. Accordingly, for 7.5 min, the penetration depth was 6.0 ± 0.1 mm (vs 4.8 ± 0.1 mm for the 5 min treatment, *p < 0.011), while for 10 min, it was 7.5 ± 0.1 mm (vs 4.8 ± 0.1 mm for the 5 min treatment, ****p < 0.0001). All these results prove that the ROS produced following the APPJ treatment of the MPC samples do not stop at the surface but interact with deeper regions.

In the second experiment with the KI-starch gels, the surfaces of the gels placed in the Petri dishes were also treated by the APPJ system for 5, 7.5, or 10 min (Figure 4B). The diameter of the navy blue colored regions observed in the case of the 1.2% agar significantly increased with the increasing treatment time only for 7.5 min (26.1 ± 0.2 mm vs 25.2 ± 0.2 mm for the 5 min treatment, *p < 0.04). In the case of the 2.0% agar, the diameter of the resultant colored regions increased with the APPJ treatment time: for 7.5 min, it was 26.2 ± 0.4 mm (vs 23.2 ± 0.5 mm for the 5 min treatment, *p < 0.01), while for 10 min, it was 28.6 ± 0.4 mm (vs 23.2 ± 0.5 mm for the 5 min treatment, *p < 0.01). The diameter of the colored regions in each case exceeded 20 mm, which was considered as the value sufficient to cover the whole surface of the treated MPC samples at each treatment time. Based on the conducted experiments, it was confirmed that ROS are generated during the MPCs treatment.

### 3.3 Characterization of Plasma Treated Cements

**Figure 5:**
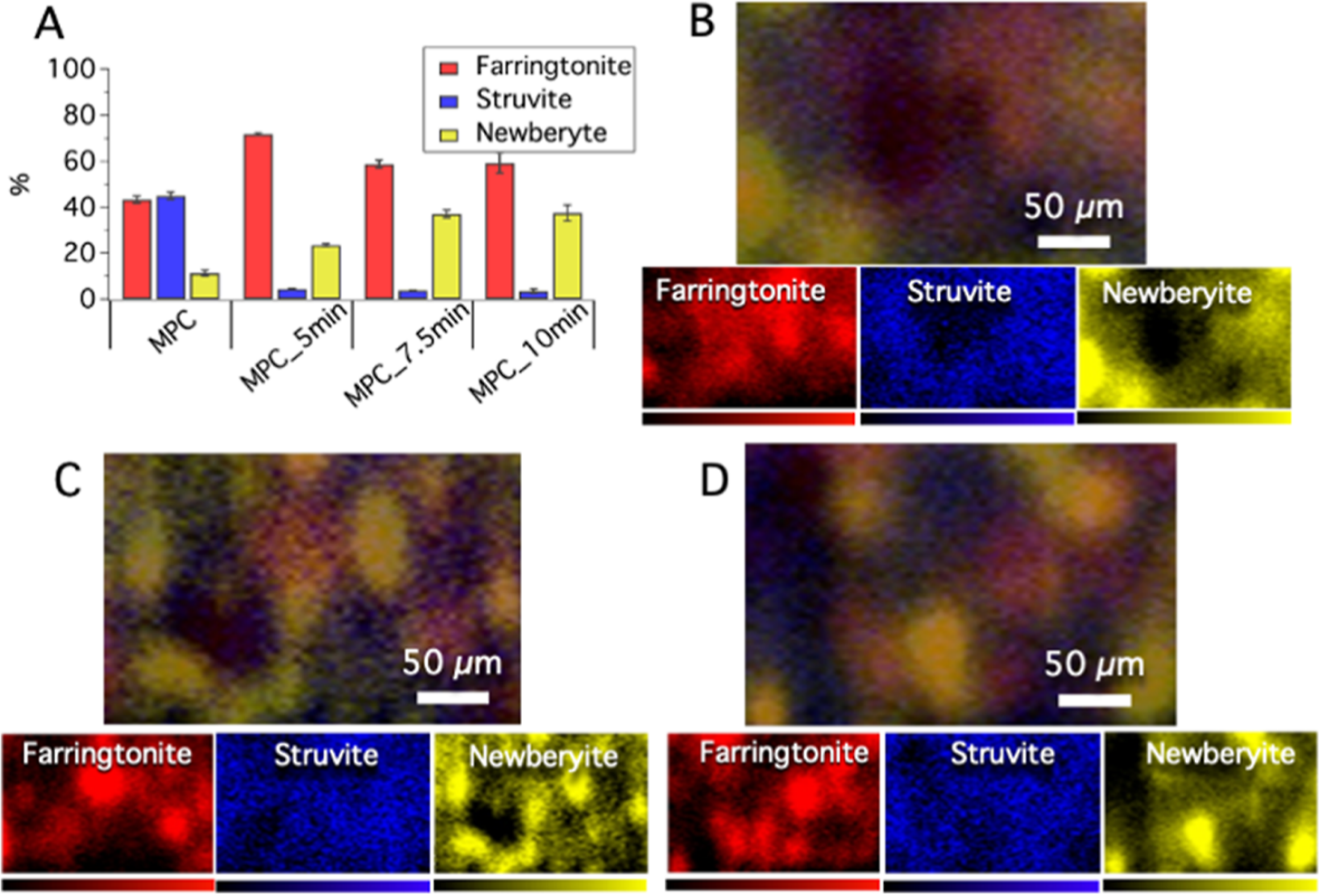
(A) Concentration estimates of the phases, calculated through the component analysis method performed on different mapped areas; confocal Raman maps of (B) MPC_5 min, (C) MPC_7.5 min, and (D) MPC_10 min, resulting from the overlay of the maps of the phases constituting the sample (farringtonite in red, struvite in blue, and newberyite in yellow). The concentration estimates and the Raman images were obtained through the multivariate method component analysis with the software WiRE, using as references the spectra acquired on the pure farringtonite, newberyite, and struvite phases.

To understand the effect of plasma on the surfaces of the investigated cements, MPCs were further characterized after the CAPP treatment by means of confocal Raman mapping. Figure 5 shows the results of the phases’ quantification performed according to the procedure detailed in Section 2.5.1, together with some representative maps collected on treated MPCs. It is evident that the plasma modifies the composition of the samples, as the amount of struvite is dramatically reduced on the surface of the treated MPCs, while the amounts of farringtonite and newberyite increase (see Table 2 and Figure 5A). As a matter of fact, struvite is known to be an unstable phase, sensitive to different external factors, including heating and low-pressure conditions, and it degrades gradually losing water and ammonia molecules.^38,43,51−53^ We can infer that CAPP had an analogous effect, favoring the conversion of struvite (MgNH_4_PO_4_·6H_2_O) to farringtonite Mg_3_(PO_4_)_2_ and/or newberyite (MgHPO_4_·3H_2_O), through the loss of some H_2_O and NH_3_ molecules upon the treatment. According to the results (Figure 5A), most of the struvite disappears in the first 5 min of CAPP treatment and then by further treating the samples some rearrangement between farringtonite and newberyite phases occurs. It is reasonable to suppose that a few minutes of the CAPP treatment (MPC_5 min) causes struvite to lose ammonia and water molecules, favoring the increase of farringtonite. It was already reported that the struvite decomposition occurs by the loss of five water molecules and subsequently loss of one water molecule during heating.^52^ In analogous conditions, it is reasonable to hypothesize that upon the CAPP treatment, some water molecules were removed from struvite and subsequently transferred through the formation of newberyite, leading to an increase of the newberyite amount in samples MPC_7.5 min and MPC_10 min with respect to the sample MPC_5 min. Moreover, according to the results, extending the plasma treatment from 7.5 to 10 min does not significantly affect the concentration of the phases, suggesting that a longer treatment would not impact the composition of the sample. For this reason, we decided to focus our attention only on MPCs treated for 5 and 7.5 min. It is worth mentioning that a local modification of the phases on the MPCs surfaces might affect their interaction with cells: out of note, in the literature some differences in terms of the osteoblasts response toward different magnesium phosphate phases are reported. An interesting work from Ewald et al. compared the cells behavior on farringtonite- and struvite-based macroporous foams, showing that the proliferation and the cell activity of the osteoblasts were higher for the farringtonite foams with respect to the struvite ones.^22^ Cao et al. observed in 3D-printed MPC scaffolds based on newberyite and struvite that MC3T3-E1 osteoblast cells could attach and spread better on the newberyite surface rather than on the struvite one.^54^ Those works suggest that the observed increase in the samples of the farringtonite/newberyite amount to the detriment of struvite due to the CAPP treatment might be an effective strategy to locally modify the surface composition and eventually favor the osteoblast response, which is propaedeutic to the new bone formation.

To understand how these phases are distributed within the surface of the samples at the microscale, Figure 5B–D shows the confocal Raman maps acquired on treated MPCs, exposing a mixture of farringtonite, struvite, and newberyite. The white light images of the mapped area are also reported in Figure S2 as comparison, coupled with the corresponding Raman maps. All treated samples display some micrometric structures of segregated phases, and we can recognize elongated objects of tens of micrometers, mainly containing newberyite.

**Figure 6:**
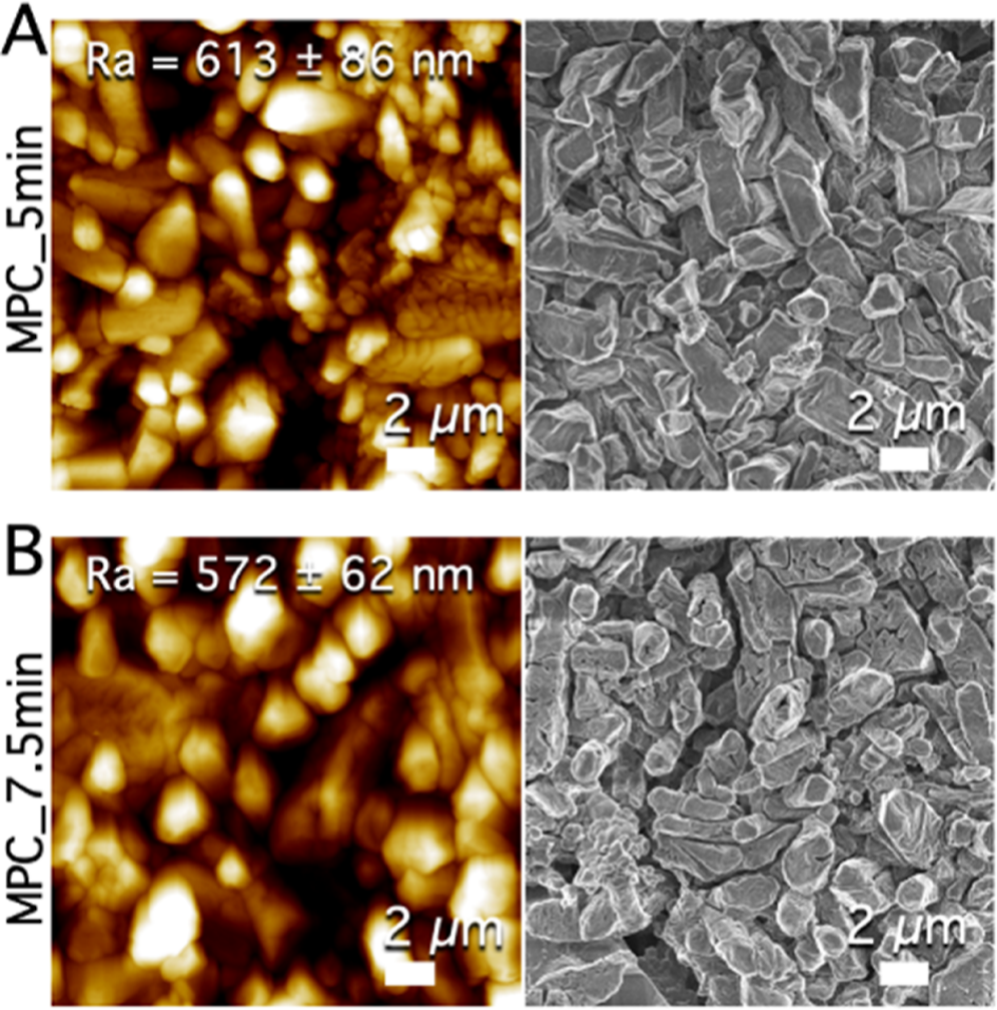
AFM maps (left) and FE-SEM images (right) of the treated cements MPC_5 min (A) and MPC_7.5 min (B), where the reported Ra values are the averages of 10 maps acquired on different regions of the samples ± standard deviations.

Figure 6A,B shows AFM maps and FE-SEM images of MPC_5 min and MPC_7.5 min, respectively. The samples display similar topography and morphology, but the surfaces expose slightly different roughness. The arithmetic mean roughness values of the investigated samples are also reported in Table 2, and it is evident that CAPP treatment slightly reduced the roughness of MPC, while significantly decreased the standard deviations associated with the roughness values. This latter evidence suggests that along the surface of the MPCs, a lower variation of the roughness values is observed upon plasma treatment. Looking at the FE-SEM images (Figure 6), we can also recognize smooth TMP crystals, while struvite crystals, characterized by prismatic cracked elongated structures, are hard to spot, confirming that in these samples struvite is present only in traces, as already evidenced by confocal Raman mapping. More FE-SEM images, collected at different magnifications, are also reported in Figure S3. We can see that the abundant prismatic elongated structures with cross-shaped and Y-shaped cracks present in the untreated MPC sample disappear after the plasma treatment in the MPC_5 min and MPC_7.5 min samples. At the same time, after the treatment, flat objects of tens of micrometers appear (see Figure S3). These structures are compatible with newberyite crystals observed in the confocal Raman maps (Figure 5), which evidence the presence of newberyite in similar elongated structures of tens of micrometers.

### 3.4 Applications

#### 3.4.1 SBF Test

**Figure 7:**
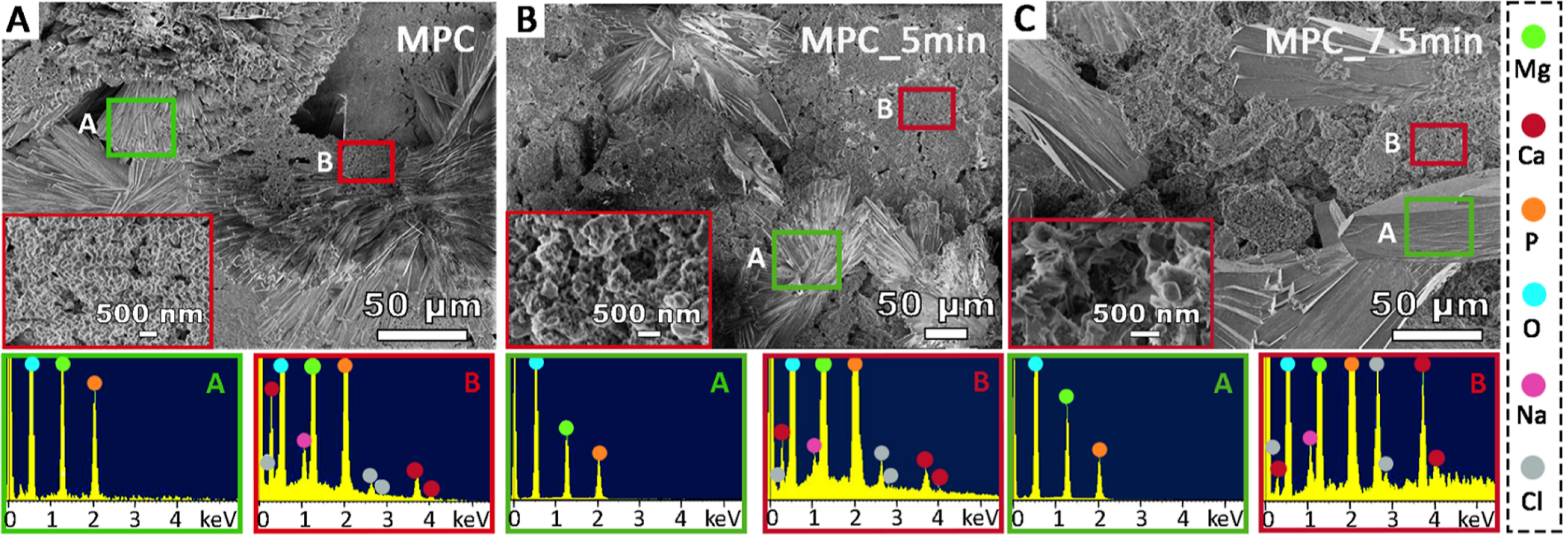
FE-SEM/EDX results of the SBF test for (A) MPC, (B) MPC_5 min, and (C) MPC_7.5 min. In each panel, SEM micrographs are given on the top, and EDX spectra on the bottom. (A) and (B) rectangles on the micrographs refer to the areas analyzed by EDX. In the insets outlined in red, high-magnification micrographs of the Ca-containing regions are reported.

The bioactivity (ability to stimulate bone growth through formation of a bone-bonding layer of apatite on the surface)^55^ of the MPCs was assessed by incubating them in SBF and evaluating the formation of calcium phosphates on the plasma-treated surfaces after 28 days of incubation. This procedure is commonly used as a preliminary test for biological experiments, as the ability of a biomaterial to support apatite formation can give information on its in vivo bone-bonding ability.^42,56,57^ The test was carried out as described in Section 2.4, and the samples were analyzed by means of FE-SEM and EDX (details in Section 2.5.3). The obtained results are shown in Figure 7 and in Figure S5. All samples incubated in SBF show large flower-like crystals deposited on the surface of the MPCs, which have a size of tens of μm. In addition, smaller objects with irregular morphologies are also present both within the crystals and on the cement surface (see the insets in Figure 7A–C). The two different regions were analyzed by EDX, to find out their semiquantitative elemental composition, both through the acquisition of spectra (Figure 7) and by elemental mapping analyses (Figure S5). In all the samples, we found that the large flower-like crystals (regions outlined in green and marked with A in Figure 7) are constituted by Mg, P, and O, while the smaller structures (regions outlined in red and marked with B in Figure 7) also reveal the presence of Ca signals. The calcium elemental mapping (see Figure S5) further confirmed the abundance of Ca on the cement surface, mostly concentrated on the small irregular objects covering the surface of all the investigated samples, while it was not detected on the magnesium phosphate flower-like crystalline structures. Therefore, the surfaces of both untreated and plasma treated MPCs support the formation of calcium phosphates. To understand the origin of the large magnesium phosphate-based crystals formed on all MPCs surfaces (green regions in Figure 7, marked with A), we incubated an untreated sample in water for 28 days at 37 °C (“Control” sample) and imaged its surface with FE-SEM. The obtained micrographs (see Figure S6) reveal that also the incubation in water leads to the formation of such structures, which we hypothesize are due to slow dissolution and reprecipitation processes occurring on the surface of the cement. Calcium is, in fact, present only in the small and irregular objects deposited on the MPCs surfaces upon incubation in SBF, as shown in Figure S5 and in Figure 7 (red regions marked with B). Out of note, the morphology of the calcium phosphate containing objects resemble an amorphous material rather than a crystalline one, possibly due to the presence of Mg on the MPC surfaces that is well-known to inhibit the crystallization of calcium phosphates.^58^ The formation of an amorphous calcium phosphate rather than a well crystalline one might be considered an advantage in this context, as amorphous phosphates display a higher solubility and can be more easily remodeled by bone cells to leave room for biogenic apatite. In summary, plasma treatment does not hinder the ability of MPCs to support the formation of calcium phosphates on their surface, suggesting their potential bioactivity when they are used as scaffolds for orthopedic applications.

#### 3.4.2 Biocompatibility of MPCs with Human Mesenchymal Stem Cells of Adipose Tissue-Origin

**Figure 8:**
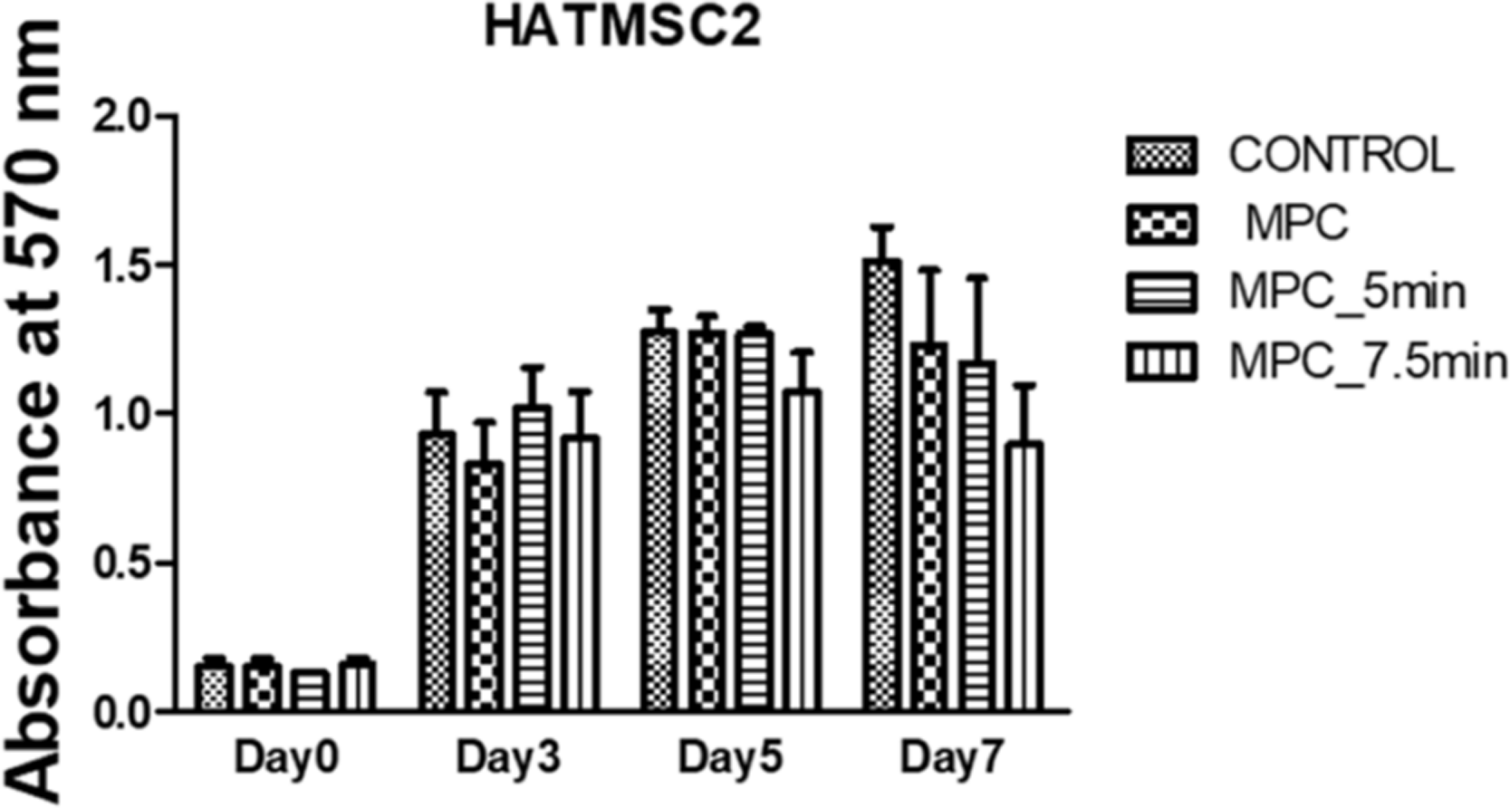
Proliferation was measured as the absorbance at 570 nm of HATMSC2 cells cultured in the presence of untreated MPC scaffold and APPJ-treated scaffolds (MPC_5 min and MPC_7.5 min). As a control, cells cultured in the culture medium without MPC scaffolds were used. The data are given as the mean ± SEM values for two independent experiments performed in duplicate.

Bioactivity of MPCs is one of the most significant factors which allows the cells to be attracted and creates a favorable environment when applied in vivo. However, to achieve the desired effects a biocompatibility with human cells is crucial to maintain cell proliferation and differentiation into specific tissue. Thus, the biocompatibility of human MSCs of adipose-tissue origin (HATMSC2) with MPC scaffolds has been tested in vitro for cell proliferation, attachment, and osteogenic differentiation. HATMSC2 cells cultured in the presence of MPC scaffolds, either untreated or APPJ-treated (MPC_5 min and MPC_7.5 min), do not inhibit cell growth compared to cells kept in a culture medium (control). The higher rate of proliferation at day 7 in the control group is a result of cell culture in 2D conditions (without MPC): when adding MPC to the cell culture, a 3D culture model is created and part of the cells attach to the scaffold and are not detectable with this method. The results are shown in Figure 8.

**Figure 9:**
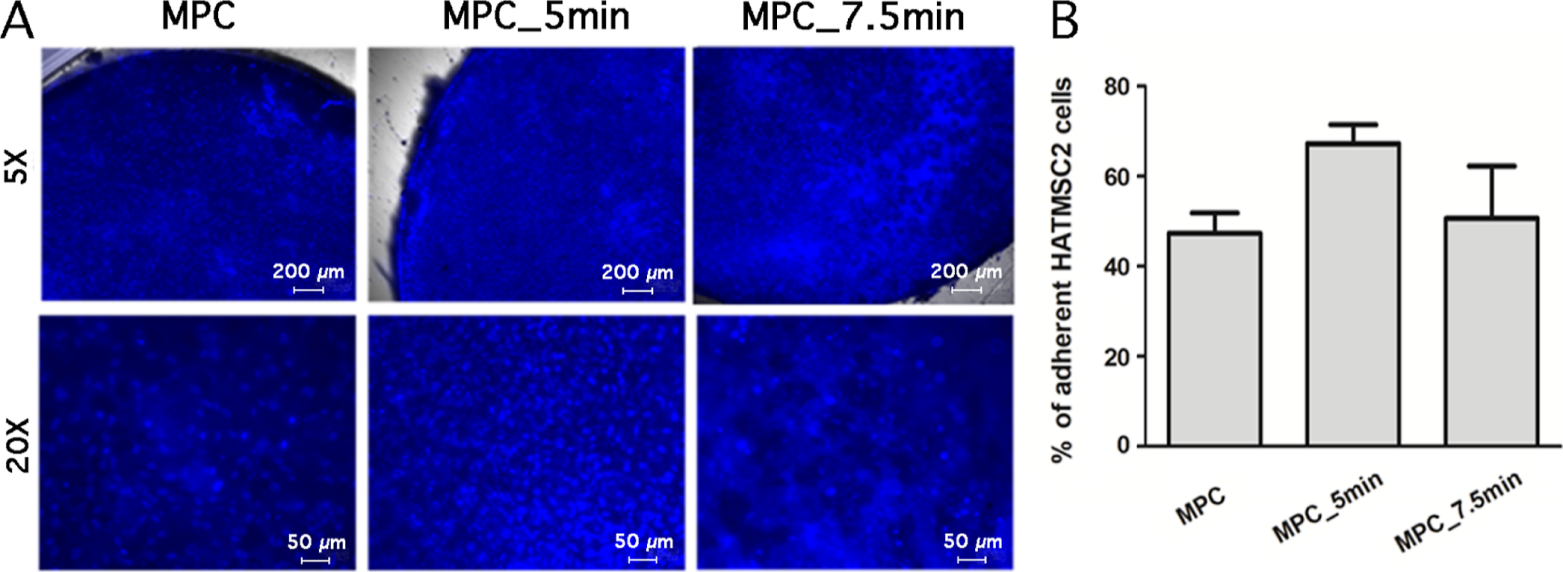
Adhesion ability of HATMSC2 cells to APPJ-treated MPC scaffolds. (A) Representative images showing the number of adhered cells to untreated MPC- and APPJ-treated MPC scaffolds for 5.0 and 7.5 min. The images of Hoechst 33342-positive cell nuclei documented the presence of live cells; the scale bar represents 200 and 50 μm, respectively. (B) Percentage of adherent HATMSC2 cells on the untreated and APPJ-treated MPC scaffolds verified by PicoGreen staining and spectrophotometric quantification. The data are given as the mean ± SEM values for two independent experiments performed in duplicate.

Cell adhesion to scaffolds is crucial for assembling biological functional constructs that restore damaged tissues. To confirm the ability of cell adhesion suggested by the results of the MTT assay, the ability for adhesion of HATMSC2 cells to MPC scaffolds has been evaluated based on the microscopic assessment and PicoGreen protocol. It was shown that HATMSC2 cells adhere to APPJ-treated scaffolds (MPC_5 min and MPC_7.5 min) as well as to the untreated scaffold (MPC) as confirmed by the presence of numerous live cells identified by Hoechst 33342-positive cell nuclei staining (Figure 9A). However, the PicoGreen assay revealed that MPC treated with CAPP for 5 min has better adhesion properties compared to MPC APPJ-treated for 7.5 min or untreated MPC (67.2 ± 4.2% vs 50.6 ± 11.6% vs 47.3 ± 4.5%, respectively, see Figure 9B).

**Figure 10:**
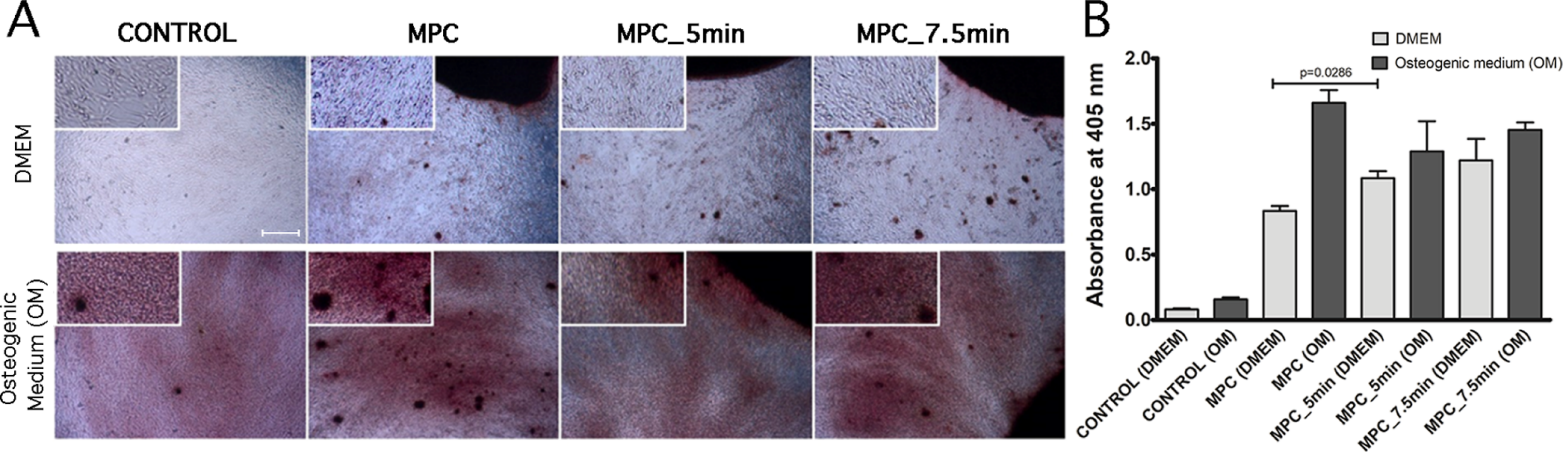
Osteogenic differentiation of HATMSC2 cells assessed by Alizarin Red S staining in the absence of MPC scaffold (control) and in the presence of MPC scaffolds both untreated and APPJ-treated. (A) Representative microscopic images showing the results of osteogenic differentiation by calcium deposit detection. The mineralized extracellular matrix was stained with Alizarin Red S dye. Scale bar represents 500 μm (magnification 4×); a higher magnification (10×) is marked with the frame; (B) spectrophotometric quantification of Alizarin Red S staining of HATMSC2 growing on the MPC scaffolds, using the cetylpyridinium chloride extraction method, measured as the absorbance at 405 nm. Alizarin Red S was quantified in two independent experiments performed in duplicate.

Finally, to assess the osteogenic differentiation potential of HATMSC2 in the presence of MPC, the cells were cultured in the osteogenic differentiation media. The results of osteogenic differentiation of HATMSC2 cells revealed that untreated MPC or APPJ-treated either 5.0 or 7.5 min do not affect the osteogenic potential of HATMSC2 cells cultured in an osteogenic differentiation medium compared to the control (Figure 10A). Quantification of Alizarin Red S staining of HATMSC2 growing on the MPC scaffolds revealed that the highest impact on osteogenesis of HATMSC2 has been observed on untreated MPC compared to the APPJ-treated for 5.0 and 7.5 min and cultured in osteogenic differentiation medium (absorbance at 405 nm 1.66 ± 0.1 vs 1.3 ± 0.22 vs 1.45 ± 0.06, respectively; see Figure 10B). It is worth noticing that HATMSC2 cultured in control DMEM medium (without osteogenic stimulatory factors) in the presence of MPC also entered in the osteogenic differentiation process as confirmed by the detection of calcium deposits in microscopic assessment; however, this process was less efficient compared to culture in the osteogenic medium. Moreover, MPC APPJ-treated for 5.0 and 7.5 min revealed higher rate of bioactivity for HATMSC2 cultured in the control DMEM medium compared to untreated MPC, as documented by the quantification of Alizarin Red S staining of HATMSC2 seeded on MPC (absorbance at 405 nm 1.08 ± 0.05 vs 1.22 ± 0.16 vs 0.83 ± 0.04, respectively), and significant difference has been observed between untreated MPC and MPC treated for 5.0 min (*p* = 0.0286). All culture conditions, either DMEM or osteogenic medium, significantly increased osteogenic differentiation of HATMSC2 in the presence of MPC compared to the controls (see Figure S7). These observations, with high probability, revealed that the physicochemical properties of MPCs favor osteogenic differentiation of HATMSC2 without the additional stimulus with an osteogenic medium containing trophic factors facilitating osteogenesis. Moreover, the APPJ-treatment of MPCs supports the efficiency of osteogenic differentiation but does not hamper the proliferation of cells in the studied model.

## 4 Conclusions

Nowadays, the use of plasma to improve the features of biomaterials is well-established: plasma not only modifies their wettability and surface properties but also contributes to their sterilization and bioactivity. In this context, our goal was to study the effect of CAPP treatments on MPCs, which are gaining importance as solutions to prepare bone cements and scaffolds. MPCs were prepared by mixing TMP with aqueous solutions of DAHP. The surface of set cements was treated with APPJ for 5, 7.5, and 10 min, and the RONS generated during the process were determined with OES. A model system was used to estimate depth and size of the generated APPJ, showing that the treatment extends to a depth of a few millimeters and has a spatial distribution of about 3 cm, depending on the treatment time. The phase composition of the MPC surfaces was studied with confocal Raman microscopy showing the distribution of struvite, newberyite, and farringtonite crystalline phases in the untreated and treated samples, finding that APPJ produces a dramatic decrease in the amount of struvite. We can infer that APPJ treatment results in partial removal of NH_3_ and H_2_O molecules from struvite, known to be a highly unstable phase, leading to the transformation into newberyite and farringtonite. FE-SEM and AFM were used to investigate the morphology and roughness of MPCs: a slight decrease in both roughness values and the associated standard deviations was found when increasing treatment time, suggesting a homogenizing effect of APPJ on the surfaces.

As a preliminary test for bioactivity, MPCs were incubated in SBF and FE-SEM/EDX experiments revealed that all samples support the formation of calcium phosphate, suggesting in vivo bioactivity of the materials. Biological experiments showed that MPCs, either untreated or treated with CAPP, are biocompatible with the examined HATMSC2 cells, as proved by their proliferation activity, adhesion efficacy, and ability to differentiate HATMSC2 into cells with osteogenic potential. These studies also proved that CAPP treatments increase the biological activity of MPC scaffolds in terms of the osteogenic stimulus. Overall, we demonstrated that MPCs, already proven as injectable and moldable materials,^15,40^ preserve a good biocompatibility upon CAPP treatment despite modifying their phase composition. As a future perspective, the comparison of the effect of CAPP toward MPCs vs CPCs would also be of great interest to further demonstrate the potential of such materials and the use of CAPP treatments on them.

Here, we unraveled the effect of plasma on already set MPCs, envisaging those applications where cements are designed to be prepared, shaped, and hardened well in advance before their application, while the CAPP treatment immediately precedes the implantation. Future developments could involve the study of CAPP treatments on the cement pastes during their setting, also taking advantage of its sterilizing effect and the potential integration into miniinvasive surgical tools. In this perspective, it would be crucial to take into account the effect of CAPP toward the healthy tissue surrounding the cement. The response of tissue cells to the direct or indirect CAPP treatment depends on their type; it is dissimilar for prokaryotic organisms and eukaryotic organisms. In the case of animal and human cells, it usually stimulates their viability and enhances their proliferation, differentiation, and migration. On the other hand, relatively long exposures to the CAPP treatment typically induce the apoptosis of such cells. Considering the internal tissues treatment, the controlled direct or indirect CAPP treatment leads to the increased production of ROS and RNS by both healthy and tumor cells due to changes in their antioxidant systems. Fortunately, this increases the proliferation activity of the tumor cells and causes their apoptosis because the healthy cells rather tolerate such increase. In addition, the difference in the membrane lipid structure of both types of cells, i.e., healthy and tumor, even facilitates the immunological death of cancer cells, leaving the healthy cells with no significant side effects.

## Supporting Information Available

Raman spectra of the pure phases, white-light and Raman images of the MPCs, FE-SEM images of the cements, OES spectra of APPJ, EDX Ca mapping of the MPCs incubated in SBF, FE-SEM/EDX images of MPC incubated in water, and spectrophotometric quantification of Alizarin Red S staining (PDF)

## Notes

Patents: The construction of the CAPP-based system, generating APPJ, is protected by Polish Patent no. P 241305 (UP RP).

## Abbreviations

AFM: atomic force microscopy
APPJ: atmospheric pressure plasma jet
CAPP: cold atmospheric pressure plasma
DAHP: di-ammonium hydrogen phosphate
DBD: dielectric barrier discharge
EDX: energy-dispersive X-ray spectroscopy
FE-SEM: field emission scanning electron microscopy
FVB: full vertical binding
HATMSC2: human adipose tissue mesenchymal stem cell line
MPC: magnesium phosphate cement
OES: optical emission spectrometry
ROS: reactive oxygen species
RONS: reactive oxygen and nitrogen species
SBF: simulated body fluid
SEM: standard error of mean
TMP: tri-magnesium phosphate
