# Supplementary material for: Effect of Atmospheric Pressure Plasma Jet Treatments on Magnesium Phosphate Cements: Performance, Characterization, and Applications
Source: ACS Biomater Sci Eng. 2023 Nov 20;9(12):6632–43. doi: 10.1021/acsbiomaterials.3c00817 (PMC10716815; doi:10.1021/acsbiomaterials.3c00817)
Supplement: Supplementary file 1 — ab3c00817_si_001.pdf [file ab3c00817_si_001.pdf]

Supporting Information

Effect of Atmospheric Pressure Plasma Jet treatments on Magnesium Phosphate Cements: performance, characterization, and applications

Rita Gelli[a], Monica Tonelli[a], Francesca Ridi[a], Dominik Terefinko[b], Anna Dzimitrowicz[b], Pawel Pohl[b], Aleksandra Bielawska-Pohl[c], Piotr Jamroz[b,*], Aleksandra Klimczak[c,*], Massimo Bonini[a,*]

[a] *Department of Chemistry "Ugo Schiff" and CSGI, University of Florence, via della Lastruccia 3, 50019 Sesto Fiorentino, Florence, Italy;*
rita.gelli@unifi.it (R.G.); monica.tonelli@unifi.it (M.T.); francesca.ridi@unifi.it (F.R.); massimo.bonini@unifi.it (M.B.).

[b] *Wroclaw University of Science and Technology, Faculty of Chemistry, Department of Analytical Chemistry and Chemical Metallurgy, 27 Wybrzeze Wyspianskiego, 50-370 Wroclaw, Poland; anna.dzimitrowicz@pwr.edu.pl (A.D.); dominik.terefinko@pwr.edu.pl (D.T.); pawel.pohl@pwr.edu.pl (P.P.); piotr.jamroz@pwr.edu.pl (P.J.)*

[c] *Hirszfeld Institute of Immunology and Experimental Therapy, Polish Academy of Sciences, The Laboratory of Biology of Stem and Neoplastic Cells, 12 R. Weigla, 53–114 Wroclaw, Poland; aleksandra.bielawska-pohl@hirszfeld.pl (A.B.-P.); aleksandra.klimczak@hirszfeld.pl (A.K.)*

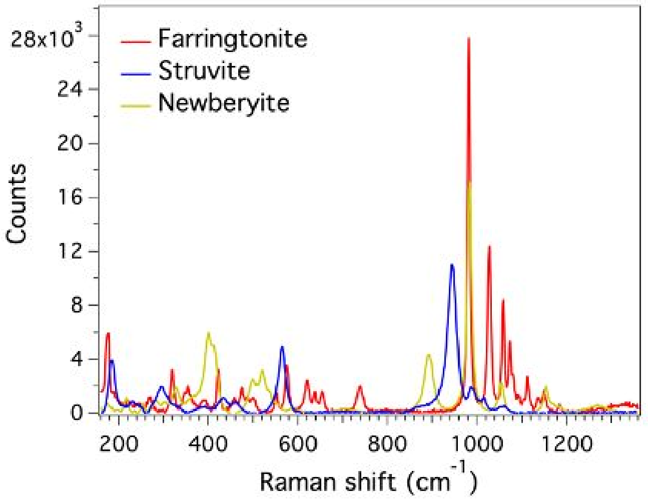

Figure S1. Raman spectra of pure farringtonite, struvite and newberyite phases.

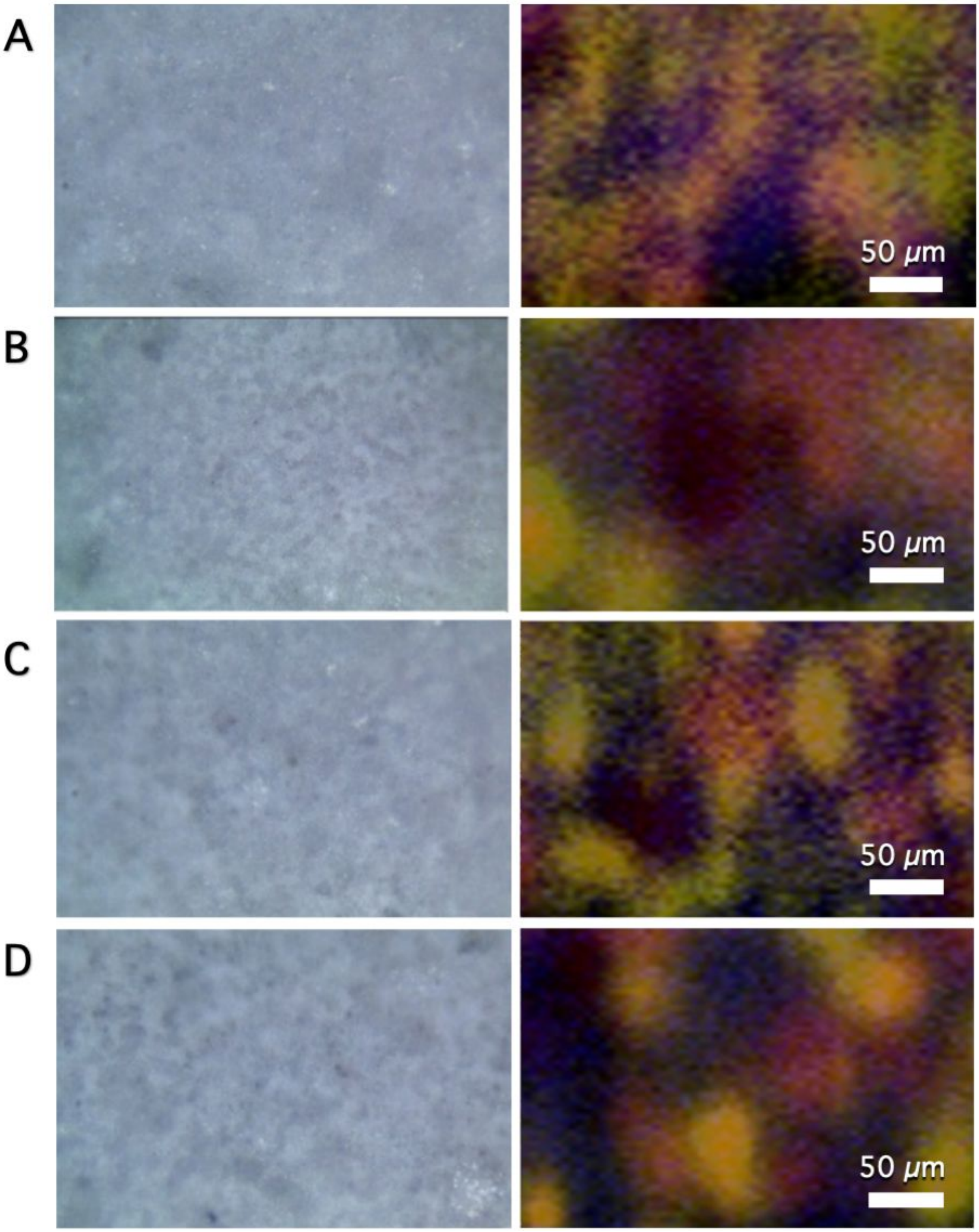

Figure S2. White light (left) and Raman images (right) of (A) untreated MPC, (B) MPC_5min, (C) MPC_7min, (D) MPC_10min samples. The Raman images result from the overlay of the maps of the phases constituting the sample (farringtonite in red, struvite in blue and newberyite in yellow).

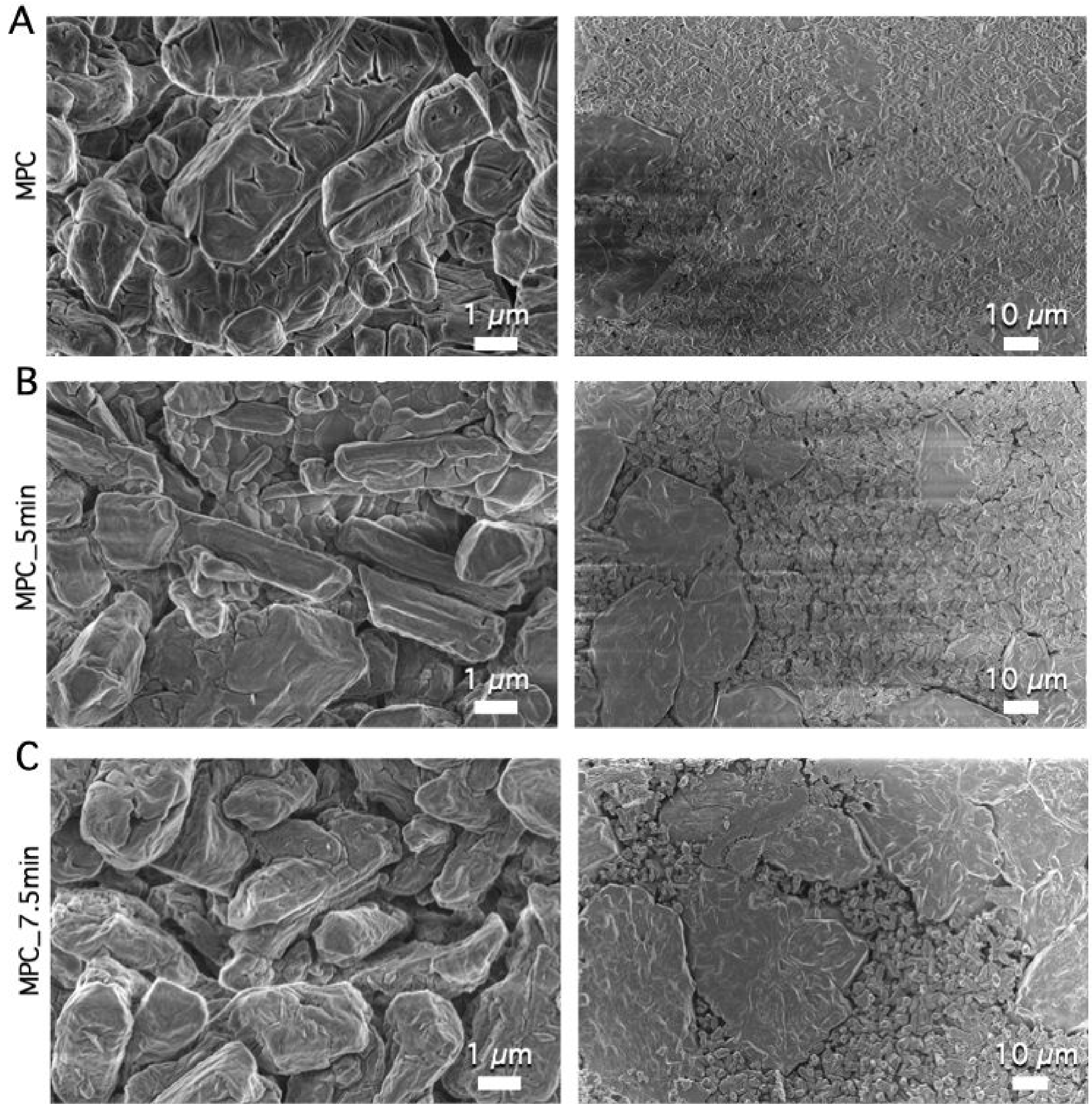

Figure S3. FE-SEM images the untreated MPC (A) and treated cements MPC_5min (B) and MPC_7.5min (C).

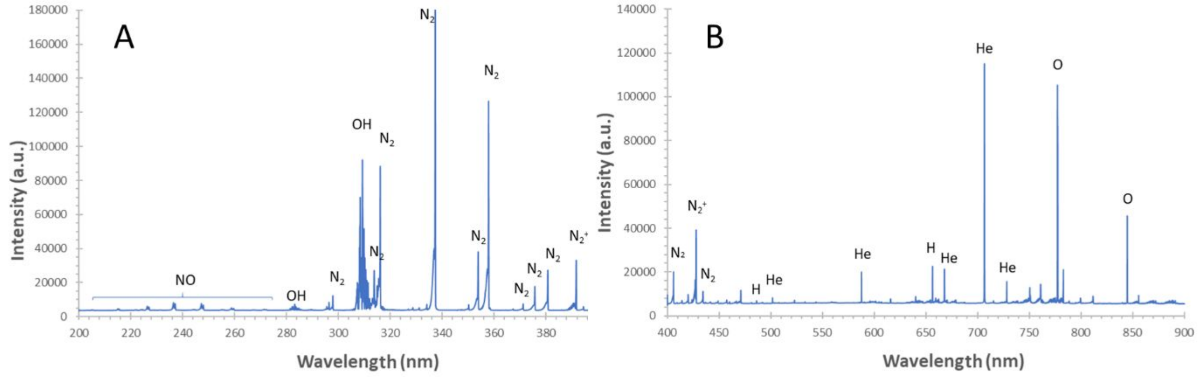

Figure S4. The OES spectra of APPJ, recorded in the (A) 200-400 nm and (B) 400-900 nm spectral ranges.

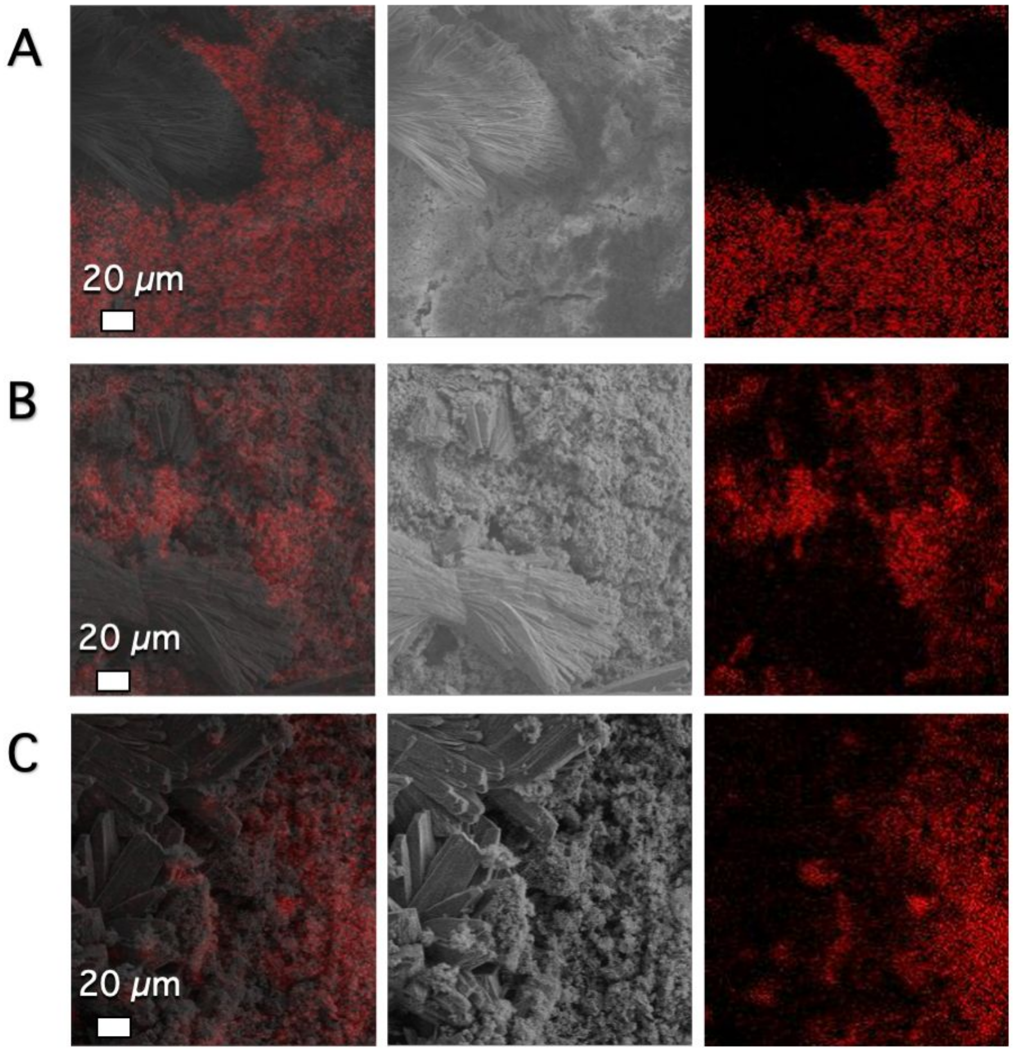

Figure S5. FE-SEM/EDX analysis (left), FE-SEM micrographs (center) and Ca elemental mapping (right) of the SBF test performed on (A) MPC, (B) MPC_5min, (C) MPC_7.5min samples.

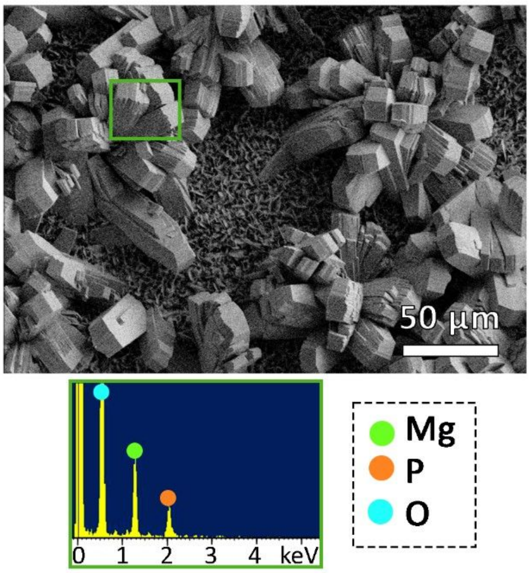

Figure S6. On the top, FE-SEM micrograph of control sample, *i.e.* MPC incubated in water at 37 °C for 28 days. On the bottom, EDX spectrum of the green region in the micrograph.

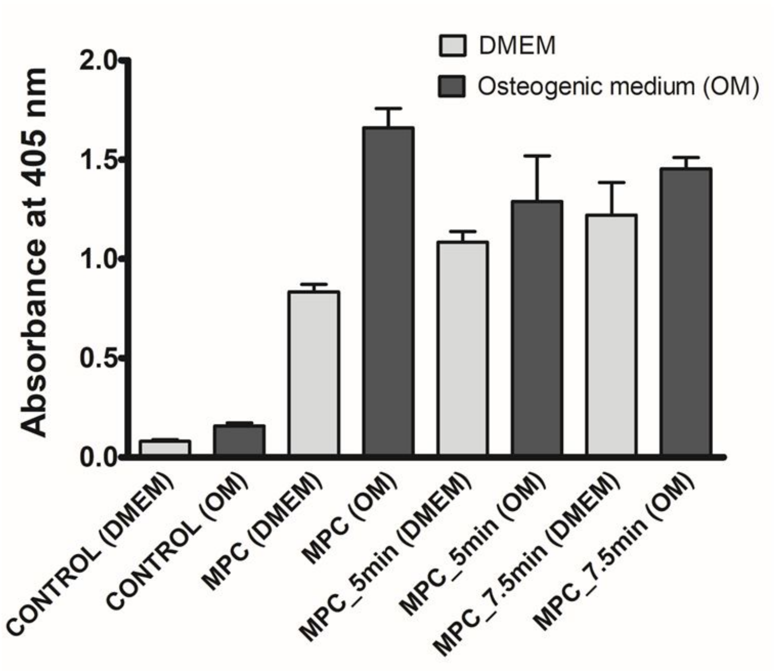

Mann Whitney test	p value
CONTROL (DMEM) vs CONTROL (OM)	**0.0286**
CONTROL (DMEM) vs MPC (DMEM)	**0.0286**
CONTROL (DMEM) vs MPC (OM)	**0.0286**
CONTROL (DMEM) vs MPC_5min (DMEM)	**0.0286**
CONTROL (DMEM) vs MPC_5min (OM)	**0.0286**
CONTROL (DMEM) vs MPC_7.5min (DMEM)	**0.0286**
CONTROL (DMEM) vs MPC_7.5min (OM)	**0.0286**
CONTROL (OM) vs MPC (DMEM)	**0.0286**
CONTROL (OM) vs MPC (OM)	**0.0286**
CONTROL (OM) vs MPC_5min (DMEM)	**0.0286**
CONTROL (OM) vs MPC_5min (OM)	**0.0286**
CONTROL (OM) vs MPC_7.5min (DMEM)	**0.0286**
CONTROL (OM) vs MPC_7.5min (OM)	**0.0286**
MPC (DMEM) vs MPC (OM)	**0.0286**
MPC (DMEM) vs MPC_5min (DMEM)	**0.0286**
MPC (DMEM) vs MPC_5min (OM)	0.2000
MPC (DMEM) vs MPC_7.5min (DMEM)	0.0571
MPC (DMEM) vs MPC_7.5min (OM)	**0.0286**
MPC (OM) vs MPC_5min (DMEM)	**0.0286**
MPC (OM) vs MPC_5min (OM)	0.3429
MPC (OM) vs MPC_7.5min (DMEM)	0.1143
MPC (OM) vs MPC_7.5min (OM)	0.2000
MPC_5min (DMEM) vs MPC_5min (OM)	1.0000
MPC_5min (DMEM) vs MPC_7.5min (DMEM)	1.0000
MPC_5min (DMEM) vs MPC_7.5min (OM)	**0.0286**
MPC_5min (OM) vs MPC_7.5min (DMEM)	0.8857
MPC_5min (OM) vs MPC_7.5min (OM)	1.0000
MPC_7.5min (DMEM) vs MPC_7.5min (OM)	0.6857

Figure S7. Spectrophotometric quantification of Alizarin Red S staining of HATMSC2 growing on the MPC scaffold, using the cetylpyridinium chloride extraction method, measured as an absorbance at 405 nm. Mann-Whitney test showed that all culture conditions, either DMEM medium or osteogenic medium, significantly increased osteogenic differentiation of HATMSC2 in the presence of MPC compared to the controls.
